# Bedside Ultrasound Conducted in Kids with distal upper Limb fractures in the Emergency Department (BUCKLED): a protocol for an open-label non-inferiority diagnostic randomised controlled trial

**DOI:** 10.1186/s13063-021-05239-z

**Published:** 2021-04-14

**Authors:** Peter J. Snelling, Gerben Keijzers, Joshua Byrnes, David Bade, Shane George, Mark Moore, Philip Jones, Michelle Davison, Rob Roan, Robert S. Ware

**Affiliations:** 1 School of Medicine and Menzies Health Institute Queensland, Griffith University, Southport, Queensland Australia; 2 Department of Emergency Medicine, Gold Coast University Hospital, Southport, Queensland Australia; 3 Sonography Innovation and Research Group (Sonar Group), Southport, Queensland Australia; 4 Child Health Research Centre, University of Queensland, Brisbane, Queensland Australia; 5 Faculty of Health Sciences and Medicine, Bond University, Gold Coast, QLD Australia; 6 Centre for Applied Health Economics, School of Medicine, Griffith University, Southport, Queensland Australia; 7 Department of Orthopaedics, Queensland Children’s Hospital, South Brisbane, Queensland Australia; 8 Paediatric Critical Care Research Group, Child Health Research Centre, The University of Queensland, Brisbane, Australia; 9 Department of Emergency Medicine, Queensland Children’s Hospital, South Brisbane, Queensland Australia; 10 Emergency and Trauma Centre, Royal Brisbane and Women’s Hospital, Herston, Queensland Australia; 11 Department of Emergency Medicine, Sunshine Coast University Hospital, Birtinya, Queensland Australia; 12 Department of Emergency Medicine, Ipswich Hospital, Ipswich, Queensland Australia

**Keywords:** Ultrasound, Bedside ultrasound, Point-of-care ultrasound, X-ray, Radiography, Forearm, Distal forearm, Fractures, Buckle fracture, Diagnostic imaging

## Abstract

**Background:**

Children frequently present to the emergency department (ED) with forearm injuries and often have x-rays to determine if there is a fracture. Bedside ultrasound, also known as point-of-care ultrasound (POCUS), is an alternative diagnostic test used to rapidly diagnose a fracture at the time of examination, without exposing children to ionising radiation. Prospective studies have demonstrated high agreement between POCUS and x-ray findings. However, whether the initial imaging modality affects the patient’s medium-term physical function is unknown.

**Methods:**

This is a multicentre, open-label, non-inferiority randomised controlled trial conducted in Australian EDs. Recruitment will continue until 112 children with distal forearm injuries (including 48 buckle fractures) per trial arm have achieved the primary outcome measure. Patients aged 5–15 years presenting with an isolated, acute, clinically non-angulated, distal forearm injury with suspected fracture will have their initial diagnostic approach randomised to either POCUS, performed by a credentialled practitioner, or x-ray imaging. If a cortical breach fracture is identified on POCUS, the patient will receive x-rays and have usual care. If a buckle fracture is identified, the patient will have their forearm placed in a splint and be discharged home. Patients will be followed up at 1, 4 and 8 weeks. The primary outcome is upper limb physical function at 4 weeks, as determined by the Pediatric Upper Extremity Short Patient-Reported Outcomes Measurement Information System (PROMIS) tool. Secondary outcomes include healthcare costs, satisfaction, pain, complications, rates of imaging, ED length of stay and diagnostic accuracy.

**Discussion:**

If POCUS is non-inferior to x-ray in terms of patient’s medium-term physical function, it may have an effect on overall health care resource use, including the number of x-ray performed and earlier ED discharge. Although prospective studies have confirmed the accuracy of POCUS, this will be the first RCT to assess non-inferiority of functional outcomes of POCUS to diagnose non-angulated paediatric distal forearm injuries, compared to x-ray. POCUS may be of particular importance in settings where access to x-ray imaging can be limited either during or after-hours, as it can aid the triaging and management of patients.

**Trial registration:**

Prospectively registered with the ANZCTR on 29 May 2020 (ACTRN12620000637943).

## Introduction

### Background and rationale

Forearm fractures account for almost 2% of children presenting to the emergency department (ED) in Australasia and are the second most common reason for presentation of children aged 10–14 years [1]. They represent about one third of all fractures in children [2], are mainly distal and usually occur after a fall [3]. Many are diagnosed as buckle (torus) fractures, which are unique to children due to their malleable cortex within a strong periosteum [4]. Unlike other paediatric fracture types of the distal forearm, such as bowing, greenstick, complete or physeal fractures [5], patients with buckle fractures can be consistently discharged with a wrist splint with no routine requirement for further imaging or follow-up [6].

The current reference standard to diagnose a distal forearm fracture is 2-view x-ray [7]. These x-rays convey a small dose of ionising radiation, which should be minimised where possible in the paediatric population [8]. Bedside ultrasound, otherwise known as point-of-care ultrasound (POCUS), a portable form of non-ionising imaging conducted at the patient bedside [9], has the potential to avoid unnecessary x-ray imaging in children with clinically non-angulated distal forearm fractures [10], thereby supporting the ‘as low as reasonably achievable’ (ALARA) principle [11]. POCUS has been proposed as a potential alternative to x-rays to diagnose non-angulated paediatric distal forearm fractures. Systematic reviews of diagnostic studies have found POCUS is both highly sensitive and specific but studies were heterogeneous, with a mixture of users and techniques [12–14].

POCUS could potentially be used as a screening tool to determine the presence and type of fracture [10, 15]. Patients without any fracture or with only a buckle fracture can be conservatively managed akin to soft tissue injuries [6]. However, patients with a cortical breach fracture require x-rays to determine the extent of injury, management and follow-up. If patients with either no fracture or with only a buckle fracture can be reliably distinguished from a cortical breach fracture using POCUS, x-rays can be avoided, with potential favourable effects on costs for the patient and healthcare system [16]. Such process and economic outcome benefits would be of interest if the initial diagnostic approach using POCUS would lead to non-inferior patient-centred outcomes. To date, no randomised controlled trial (RCT) has compared POCUS versus x-ray as the initial diagnostic approach in terms of functional outcomes [12–14].

### Objectives

The primary objective of this trial is to assess whether, in children presenting to ED with distal forearm fractures, initial diagnostic imaging modality (POCUS compared to x-ray) affects functional upper limb outcomes. A secondary objective includes a health economic analysis to determine the cost-effectiveness of POCUS compared to x-ray as the initial imaging. We hypothesise that when POCUS, instead of x-ray, is used as the initial diagnostic tool for paediatric distal forearm fractures, upper limb physical function is non-inferior at 28 days post injury, with an overall reduction in x-rays and costs, without missing any clinically significant fractures.

### Trial design

This is a multicentre, open-label, non-inferiority RCT comparing paediatric clinically non-angulated distal forearm injuries by initial diagnostic imaging modality (POCUS to x-ray) to assess functional upper limb outcomes. The protocol adheres to the Standard Protocol Items: Recommendations for Interventional Trials (SPIRIT) Statement.

## Methods: participants, interventions and outcomes

### Study setting

The trial will be conducted across participating hospitals in Queensland, Australia. The participating hospitals will include both tertiary and non-tertiary centres, as well as a mixture of dedicated paediatric EDs and EDs with both adult and paediatric presentations.

### Eligibility criteria

The trial will recruit children aged between 5 and 15 years (i.e. up to 15 years and 364 days) with an isolated, acute, clinically non-angulated, distal forearm injury who are being further evaluated for a suspected fracture with imaging. The population of children between the ages of 5 and 15 years was chosen as the children within this age range can all sustain buckle fractures [17]. Patients with additional injuries deemed to require imaging, including midshaft forearm, proximal forearm, scaphoid or elbow tenderness, will be excluded. Patients will need to able to attend follow-up, if required, ideally living within a ~ 50-km radius of the enrolling hospital and have telephone and Internet access. See Table 1 for full inclusion and exclusion criteria.

**Table 1:** Inclusion and exclusion criteria

Inclusion criteria	
• Child age 5–15 years *with*	
• Distal forearm injury *and*	
• Can attend any follow-up	
Exclusion criteria	
• Obvious angulation or deformity (soft tissue swelling allowed)	
• Injury sustained > 48 h prior	
• External x-rays already performed	
• Compound/open fracture (including concern for foreign body)	
• Neurovascular compromise	
• Known bone disease (e.g. osteogenesis imperfecta)	
• Suspicion of non-accidental injury	
• Additional injuries requiring x-rays (e.g. elbow, scaphoid)	
• Congenital forearm malformation (e.g. radius hypoplasia)	
• No credentialled clinician available to perform scan	
• Significant developmental delay or behavioural difficulties prohibiting accurate clinical assessment	

### Consent

Potentially eligible patients will be identified by nursing staff at triage with subsequent review by a credentialed clinician to examine patients and confirm final eligibility according to inclusion and exclusion criteria. Informed consent will be obtained from the legal guardian, hereafter referred to as the parent, after they have reviewed an information sheet. This is supplemented by an online video recording, with parents and/or participants having had the opportunity to ask any questions they need to. After fulfilment of written informed consent requirements, baseline data will be collected prior to randomisation.

### Interventions

Participants will be allocated to an initial diagnostic imaging modality of POCUS or x-ray. POCUS was chosen as the intervention for this trial as, for paediatric distal forearm fractures, it is non-invasive, accurate and well-tolerated; does not emit ionising radiation; and is preferred by patients [10]. X-ray was chosen as the control comparator as not only is it a pragmatic reference standard, but also it currently guides routine practice in the ED and subsequent standard outpatient follow-up. MRI was considered costly and impractical to use as the reference standard in an acute setting and may detect clinically irrelevant fractures or bone contusions, and CT would expose patients to unnecessary ionising radiation [18].

#### Pocus

Eligible patients randomised to POCUS will have imaging performed by a health practitioner (nurse, doctor or allied health professional) who has undergone training and credentialing. Credentialing consists of a 2-h simulated training package with a phantom arm model [16], followed by proctored scanning, the collection of a logbook of scans demonstrating a total of 20 patients with a mixture of at least 10 buckle or cortical breach fractures, image interpretation from a bank of case examples and a final observed assessment. A modified 6-view forearm POCUS protocol will be conducted, whereby the distal radius and ulna are interrogated on their dorsal, lateral and volar aspects with a high frequency linear probe in a longitudinal axis with the probe marker orientated distally [10]. Secondary signs will also be assessed (Table 2), which includes the pronator quadratus haematoma sign [19]. Patients will be provided with analgesia as required and rest their forearm, ideally on a pillow, during the procedure. Pain will be minimised by use of gel and minimal probe pressure. The final image(s) will be prospectively labelled with the overall forearm diagnosis, being as specific as possible for the cortical breach subtype.

**Table 2:** Criteria for consideration of x-ray imaging after POCUS

POCUS Primary sign	
• Identification of a cortical breach fracture (apart from an isolated ulna styloid fracture or non-displaced, non-angulated ulna metaphyseal fracture)	
POCUS Secondary signs:	
• Buckle fracture < 1 cm from physis	
• Pronator quadratus haematoma present (i.e. positive fat pad sign)	
• Angulation greater than ~ 5 degrees (visually angulated)	
• Physis widened or narrowed	
• Periosteal haematoma	
Clinical basis	
• Clinical suspicion, despite normal POCUS findings e.g. ‘pain out of proportion’ as per clinician judgement	

Patients will receive an overall forearm diagnosis based on the POCUS findings and be classified into 3 fracture categories: ‘no’, ‘buckle’ or ‘other’. A ‘buckle’ fracture is defined as an inward or outward deformation of bone cortex without breach. The ‘other’ fracture group will consist of any fracture with a cortical breach identified using POCUS. A cortical breach is defined as a break, step or gap in the bone cortex. Both the radius and ulna bones will be individually classified, with the overall forearm classification based on the overarching fracture that determined the management of the patient (Table 3). If classified as ‘other’ fracture on POCUS, they will receive an x-ray and be managed on that basis. If either ‘no’ or ‘buckle’ fracture is identified on POCUS, the patient will not receive an x-ray prior to discharge. Ulna styloid, or non-displaced, non-angulated ulna metaphyseal fractures will be considered clinically insignificant and can be managed conservatively in a wrist splint. Both the x-ray and POCUS images will be later reviewed for correct fracture classification and clinical relevance by an expert panel.

**Table 3:** Overall fracture diagnosis group classifications

‘No’ fracture	
• No fracture of the radius or ulna	
‘Buckle’ fracture	
• Buckle fracture of the distal third radius, with or without:	
- Ulna styloid fracture	
- Ulna metaphysis non-displaced/non-angulated buckle or cortical breach fracture.	
• Isolated buckle fracture of the distal third ulna	
‘Other’ fracture	
• Cortical breach fracture of the distal third radius (including greenstick, complete or physeal), with or without any ulna fracture type	
• Any isolated displaced/angulated cortical breach fracture of the ulna metaphysis	
• Fractures at other locations, e.g. proximal radius, scaphoid	
• Bowing fractures of the radius and/or ulna with or without any other fracture type	

#### X-ray

Eligible patients randomised to x-ray will have a minimum of 2 views performed by a radiographer and reported by a radiologist or radiology trainee, with both groups masked to the initial diagnostic allocation and any POCUS findings. X-ray imaging will be interpreted by the treating clinician and classified into 3 fracture categories, as per the POCUS trial arm (‘no’/‘buckle’/‘other’). A ‘buckle’ fracture is defined as an inward or outward deformation of bone cortex without breach. The ‘other’ fracture group will consist of cortical breach fracture types, including greenstick (uni-cortical), complete (bi-cortical) or physeal (Salter-Harris), and also bowing fractures and fractures at other sites (e.g. scaphoid) if incidentally not excluded on physical examination. A cortical breach is defined as a break, step or gap in the bone cortex.

#### Management

The management principles will be the same for both the POCUS and x-ray trial arms. The ‘no’ fracture group will be conservatively managed at the clinician’s discretion, as documented in the medical record. The ‘buckle’ fracture group will be managed in a wrist splint and the ‘other’ fracture group will be managed with intervention (manipulation, reduction and/or surgery) and immobilisation in a plaster cast or equivalent as required.

All patients who are diagnosed as either ‘no’ or ‘buckle’ fracture in either study arm (POCUS or x-ray) will be given a tentative appointment for ED clinical review in approximately 1 week (5–7-day convenience window) at the site they were originally managed. The parent will be contacted with a standard script prior to the appointment and the review will be cancelled if no longer required due to clinical improvement. If a clinical review is attended, the parent and practitioner will prospectively document whether they believe x-rays are required. X-rays will then only be performed if indicated by clinical suspicion, unless the parent decides against it and this is supported by the reviewing practitioner. Patients will then receive usual care as per x-ray findings, with the orthopaedic service consulted for any displaced or angulated fractures.

#### Criteria for discontinuing or modifying allocated interventions

X-ray will be performed as per usual practice, with views limited by patient tolerability. Inadequate views may require repeating, as per clinician judgement. Although it is unusual to be unable to obtain x-rays, if this occurs, reasons will be documented. POCUS will be discontinued if the patient experiences undue distress or excessive pain whilst POCUS is performed, or if adequate images are unable to be obtained, and will receive x-rays instead. If classified as ‘other’ fracture on POCUS, such as identification of a cortical breach, they will receive an x-ray and be managed accordingly. Patients may also receive x-rays based on secondary signs of a cortical breach fracture detected on POCUS or ‘pain out of proportion’ as per treating clinician judgement. Full criteria for x-ray imaging consideration after POCUS are listed in Table 2. Patients who subsequently receive x-rays will receive routine cares based on its findings.

Once the initial diagnostic test (POCUS or x-ray) has been conducted, the intervention will be considered complete. Patients will receive analgesia as required, which will be documented for comparison between diagnostic arms and subgroups. The trial has been designed to safeguard against any clinically significant fractures being missed in either study arm. The extended POCUS protocol is designed to utilise secondary signs, such as the pronator quadratus haematoma sign, which shows promise as an additional measure to avoid missing any cortical breach fractures [19]. Furthermore, the follow-up review, if required, is an appropriate timeframe to intervene for any missed fractures. All patients with a displaced or angulated fracture of significance will receive orthopaedic specialist care, including during and after the trial. Any deviation from the protocol will be noted, specifically if this changed fracture management.

### Outcomes

#### Trial endpoints

The primary outcome is upper limb physical function at 4 weeks (28 days ±3 days) as measured by the validated Pediatric Upper Extremity Short Patient-Reported Outcomes Measurement Information System (PROMIS) tool. This timeframe was chosen, as patients with buckle fractures and soft tissue injuries should have regained normal physical function by this time, compared with cortical breach fractures. Provided there are no re-injuries, 8 weeks is an accepted time frame for fracture healing in children, with recovery of function within 3–4 weeks for children with distal forearm buckle fractures [20]. The earlier follow-up period as the primary outcome is designed to detect any difference of arm function between diagnostic groups, with complications being a secondary outcome.

**Fig. 1:**
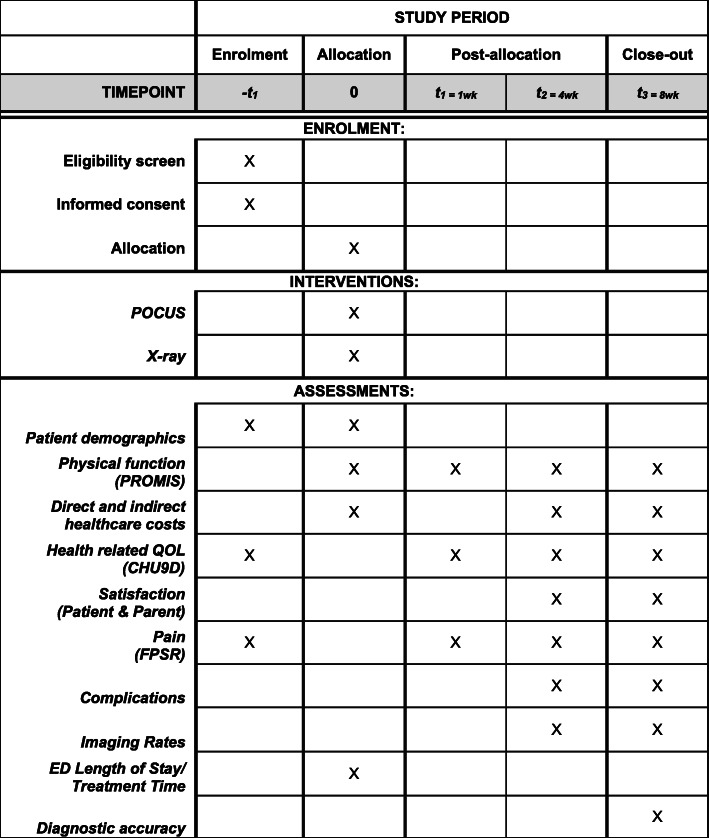
Participant timeline (SPIRIT format)

Health economic analysis will be conducted to determine the cost-effectiveness of POCUS compared to x-ray with the main outcome being the incremental cost per quality adjusted life year from both a health care sector perspective and broader societal perspective. Refer to Table 4 for complete primary and secondary outcomes. Data will be collected at designated time points across an 8-week period (Fig. 1, SPIRIT participant timeline).

**Table 4:** Outcome measures for POCUS and x-ray trial arms

Primary outcome measure:	
• Physical function of injured upper limb at 4 weeks (28 ± 3 days), as determined by the PROMIS tool.	
Secondary outcome measures:	
• Direct and indirect health care costs (healthcare provider visits, days off work/school)	
• Health related quality of life (QOL), as determined by the CHU9D	
• Satisfaction score (patient and parent)	
• Patient pain score measured using the FPSR	
• Rates of complications (alternate fracture diagnosed, poor fracture healing)	
• Rates of x-ray and other imaging (particularly the ‘no’ and ‘buckle’ fracture groups)	
• ED length of stay (triage to ED discharge)	
• Treatment time (clinician review to ED discharge)	
• Diagnostic accuracy of POCUS, including secondary signs, and x-ray	

Key: *PROMIS* Pediatric Upper Extremity Short Patient-Reported Outcomes Measurement Information System, *CHU9D* Child Health Utility 9D, *FPSR* Faces Pain Score Revised

#### Sample size

Sample size calculations were based on a non-inferiority hypothesis comparing POCUS with x-ray (routine care) for the primary outcome of upper limb function assessed using the PROMIS score at 4 weeks. Previous studies suggest the true between-group difference was assumed to be zero with the non-inferiority margin defined as an absolute difference of 5 points and the standard deviation of PROMIS assumed to be 11.5 points [21–23]. To achieve 90% power with a one-sided α of 0.025, we require primary outcome data for 224 children (112 per arm).

A key secondary endpoint is to assess the non-inferiority of POCUS compared with x-ray as the initial imaging strategy for children who are later confirmed by the expert panel to have buckle fractures. Previous studies suggest that the standard deviation of PROMIS for children with buckle fractures is approximately 7.5 points [21–23]. Assuming the true between-group difference is 0 and defining the non-inferiority margin as 5 points, then with 90% power and one-sided *α* = 0.025, we require primary outcome data on 96 children with buckle fractures (48 per arm). We assume 35–45% of participants will be diagnosed with buckle fractures by the expert panel.

Recruitment will continue until primary outcome data has been collected on both at least 112 children per arm and at least 48 buckle fractures per arm. Allowing for approximately 25% attrition and variation in buckle fracture prevalence, we expect to recruit up to 300 patients to achieve the required sample size.

#### Recruitment

To enhance completion of data collection, research staff will email, text and telephone reminders and, if necessary, will complete questionnaires over the phone with the patient. Screening logs will be kept, with a record of missed patients. We will collect non-identifiable data on children presenting to ED with forearm injuries during the study period, including those not recruited, to establish recruitment rates, determine any systematic bias and support interpretation of the economic evaluation.

#### Randomisation

Randomisation to either POCUS or x-ray as the initial diagnostic modality will be computer-generated via a web-based central randomisation service (Griffith University Clinical Trials Randomisation Service). Randomisation will occur in a 1:1 ratio within blocks of size 6–8 (size randomly selected), stratified by site and age (5–9 years and 10–15 years). Secure, password-protected web-based randomisation will occur once; the participants’ eligibility has been confirmed by the clinician and baseline data collected. Randomisation to intervention will be generated by any credentialed clinician available to perform POCUS.

#### Blinding

This is an open-label trial. However, radiographers performing the x-rays, and radiologists or trainees reporting them, will be masked to the original diagnostic allocation of patients and any POCUS images. The expert panel determining the final diagnosis will be masked to the primary and secondary outcomes.

## Data collection and management

**Fig. 2:**
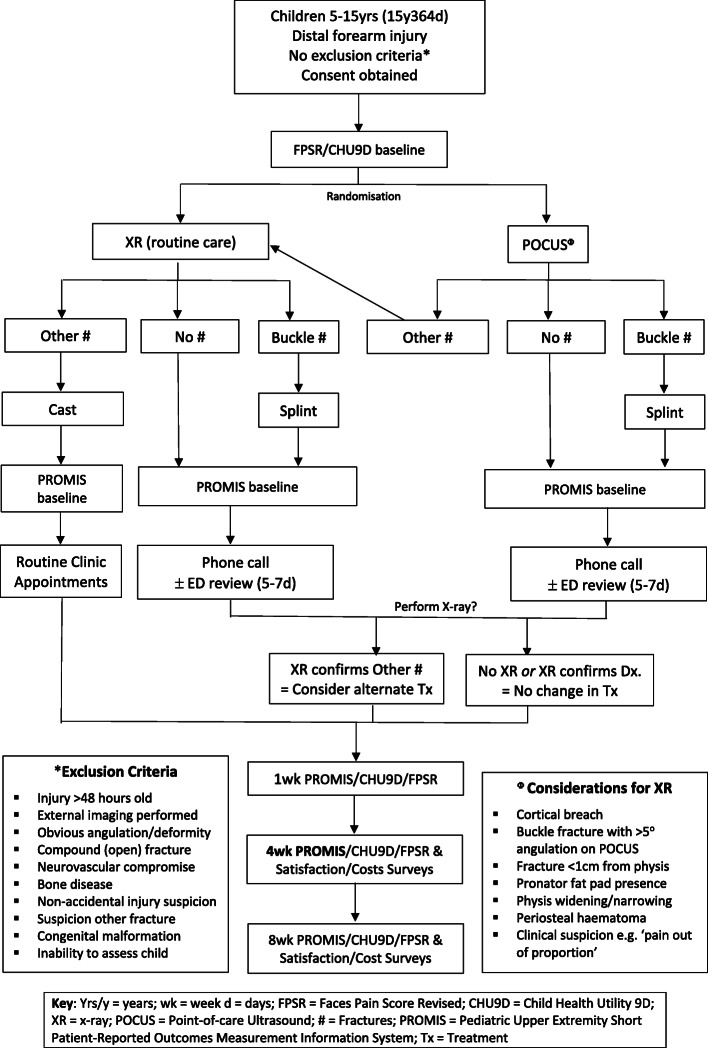
Recruitment and data collection flowchart

Data will be manually collected at the initial hospital visit, later manually entered into the secure, purpose-built web-based database, REDCap® (Research Electronic Data Capture, Vanderbilt University, Nashville, TN, USA), and then via automated electronic surveys directly linked to the database, emailed to participants after discharge at the designated timepoints, generally a combination of 1, 4 and 8 weeks (Figs. 1 and 2). If the electronic surveys have not been returned, parents will receive a phone call within 48 h to remind them to complete and submit them. A maximum of 5 attempts to contact parents or secondary contacts by either telephone, text message or email will be made by the research team. If there is no response after the 5th attempt, the participant will be considered lost to follow-up for that time point but will still be contacted at subsequent time points, unless the patient withdraws from the trial.

### Demographics

Patient demographic and clinical characteristics will be recorded, including hand preference, injured arm, mechanism of injury, time of injury, previous forearm issue (injury, surgery, dysfunction) and analgesia provided (home and in ED).

### Functional outcome

The PROMIS is a validated tool that assesses the physical function, including activities of daily living, for patients 5–17 years of age [21–23]. This tool is an 8-item survey, with each item measured on a 5-point scale. It is self-reported from 8 years old and proxy-reported (i.e. parental assistance) below 8 years, reflecting function during the previous 7 days and takes up to 10 min. It should be completed each time in the same setting, ideally in a quiet location in their own home. The duration of immobilisation, including splint or casting, will be compared between the trial arms using data from the Costs & Management Survey and the medical record.

### Health care costs

For the healthcare sector perspective analysis, the sum cost of direct healthcare resources, including implementation, staff labour, equipment and ED length of stay, will be estimated per patient for both study arms. A bottom up a costing methodology will be adopted that specifically measures resource use (i.e. number and type of diagnostics, treatment time and consumables) as well as costs associated with implementation including training. Health resource use during initial procedure will be extracted from individual electronic medical records. Data of resource use for implementation will be collected during the trial by research staff. For the societal perspective analysis, healthcare utilisation beyond the initial procedure, patient and parent time, travel and other indirect health care costs will be collected using the Cost & Management questionnaire adapted for use in this study [24]. Parent time off work and school or childcare absence, due to original injury, will be recorded for use in the societal economic evaluation. Healthcare utilisation will include return visits to the emergency department, outpatients or other practitioners, additional imaging and admission to hospital.

### Health-related quality of life

Data on health-related QOL will be gathered from the Child Health Utility 9D (CHU9D) tool [25] and scored using the Australian utility algorithm [26]. The CHU9D is a 9-item questionnaire that can be administered in less than 5 min, with self-reported from 7 years old and proxy-reported below 7 years, based on the child ‘today’. CHU9D adolescent weightings will be applied to the analysis, with estimation of quality adjusted life years for both study arms.

### Satisfaction

**Fig. 3:**
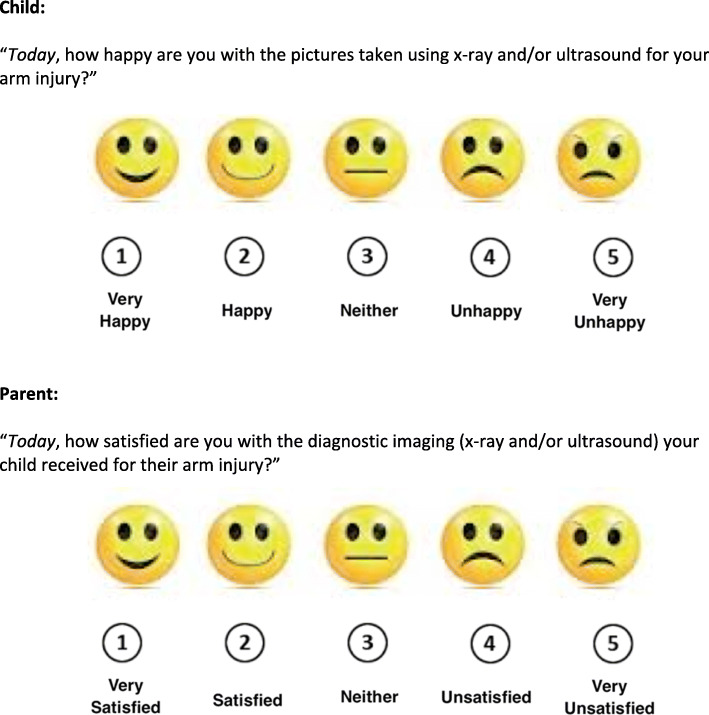
Patient and parent satisfaction rating scales

Patient and parent satisfaction scores based on a 5-point Likert scale (Fig. 3) will be collected. Patients and parents will be independently asked to rate their level of satisfaction.

### Pain

Faces Pain Score Revised (FPSR) is a reliable and validated tool for assessment of pain in children in the age range for this trial, with scores based on the patient’s current level of pain [27].

### Complications

Complications will be compared across the two trial arms and will include the diagnosis of an alternative fracture pattern to the initial diagnostic imaging, worsening fracture deformity, rates of re-injury, growth disturbance, delayed fracture healing and/or the requirement for surgical intervention. This information will be obtained from the Costs & Management survey and from the electronic medical record.

### Rates of imaging

Rates of imaging required, including x-rays, CT or MRI, will be compared between trial arms. In particular, the frequency of x-ray imaging (i.e. the number of individual x-rays performed) in the patients diagnosed with ‘no’ or ‘buckle’ fracture in each trial arm will be determined.

### ED length of stay

The length of stay (LOS) in the ED for each patient will be determined from the electronic clinical record. This will be defined from the time the patient was triaged to the time of discharge (home or admission to the ward). Treatment time will also be noted, defined as the time from clinical review of the patient until time of discharge from ED.

### Diagnostic accuracy

The diagnostic accuracy of POCUS and x-ray imaging will be determined against the gold standard final diagnosis, as determined by the consensus from an expert panel based on the clinical course, investigations and final management. The expert panel will meet regularly throughout the course of the study. A case is defined as ‘true positive’ when POCUS diagnoses ‘any’ fracture type (i.e. ‘buckle’ and ‘other’ fractures combined). A “true negative” is defined when ‘no’ fracture is identified on POCUS, matching the expert panel final diagnosis. A “false positive” is defined when ‘any’ fracture is identified on POCUS but the final diagnosis is ‘no’ fracture. A “false negative” occurs when ‘no’ fracture is found on POCUS but the expert panel identifies ‘any’ fracture. Given that buckle fractures can be managed more in keeping as a soft tissue injury, we will secondarily compare the ‘other’ fractures against ‘buckle’ and ‘no’ fracture groups combined. The same definitions will apply for the x-ray arm of the trial, for which the clinician interpretation of the x-ray at time of discharge will be compared to the gold standard diagnosis. The diagnostic utility of POCUS secondary signs and clinical findings will be evaluated.

### Ethical considerations

Once recruited, patients will be de-identified and assigned a study number linked to their hospital record number. Initial data collected by the clinician will be recorded on hardcopy case report forms, which will then be stored in a locked cabinet along with consent forms to be later entered into the electronic database. Participants will be emailed the questionnaires with direct data entry electronically captured in the password protected REDCap® database.

### Statistical methods

Summary statistics will be presented as either mean (SD) or median (25th–75th percentile) for continuous data, depending on their distribution, and as frequency (percentage) for categorical data. For the primary outcome, physical function assessed by the PROMIS tool at 4 weeks, we assess non-inferiority of POCUS within a 5-point margin. The between-group difference at 28 days will be assessed using linear regression modelling with treatment group (POCUS/x-ray) included as a main effect. Non-inferiority will be established if the 95% confidence interval for the difference between-groups is completely above the non-inferiority margin. Primary analyses will include all randomised patients. A secondary analysis of the subgroup of patients confirmed to have buckle fractures by the expert panel will be undertaken. Analysis will be on an intention-to-treat basis, with a secondary per-protocol analysis undertaken.

Secondary outcomes such as costs, satisfaction, pain, complications, rates of imaging, length of stay and diagnostic accuracy for the two study groups will be compared. Linear regression models will be used to compare continuous outcomes, logistic regression models will be used to compare dichotomous outcomes, and Poisson regression models will be used to compare count outcomes. Subgroup analyses will be performed for each category of ‘no’ fracture, ‘buckle’ fracture and ‘other’ fracture, and also for age category (5–9 and 10–15 years), given that healing rates may differ in these age groups. Missing data will not be imputed.

#### Health economic analysis

For the economic evaluation, a cost-utility analysis will be conducted from both the healthcare sector and broader societal perspective. The primary outcome will be the incremental cost per quality adjusted life year gain. From the healthcare sector perspective, only direct health care costs will be included. For the broader societal perspective costs will also include indirect costs associated with work absenteeism and travel. Analyses from both perspectives will use quality-adjusted life years as the primary measure of benefit. Supplementary analyses will also provide the cost per PROMIS unit improvement and cost per x-ray avoided. Primary calculation of quality-adjusted life years will be estimated using an area under the curve approach [28]. Secondary analysis of quality-adjusted life years will estimate change in health-related quality of life over time using multi-level regression modelling with a random intercept, including time as a repeated categorical variable and adjusting for baseline scores [29]. As costs and QALYs are usually non-normally distributed [30], we will use generalised linear regression models (a priori assuming log link and gamma error which will be tested during analysis using the Pregibon link test and modified Park’s test). The 95% confidence interval estimated using the multi-level regression approach for quality adjusted life years will be presented. Uncertainty in the incremental cost per quality-adjusted life year estimate will be assessed using bootstrapping (minimum 1000 iterations) for probabilistic sensitivity analysis with 95% credible interval being reported. The incremental cost per quality-adjusted life year will be compared against a threshold willingness to pay value of $50,000 (a commonly used threshold in Australian cost–utility studies) with the sensitivity to decision making of this threshold value assessed using a cost-effectiveness acceptability analysis [31].

#### Diagnostic accuracy

Diagnostic accuracy will be determined for ‘any’ fracture (‘buckle’ and ‘other’ fractures combined) and ‘other’ fractures alone. Diagnostic statistics including sensitivity, specificity, positive and negative predictive values and positive and negative likelihood ratios will be calculated for both POCUS and x-ray.

## Oversight and monitoring

The Gold Coast University Hospital is the BUCKLED RCT coordinating centre, with data management by the PCCRG and oversight from a steering committee of both emergency physicians and orthopaedic surgeons representing all participating hospitals. Any protocol amendments will be submitted to HREC and local governance bodies for approval before implementation. The ANZCTR will be informed of any implemented protocol amendments.

### Dissemination

The findings from this research are intended to be disseminated through local meetings, international conferences, peer-reviewed publications and websites.

## Discussion

Paediatric distal forearm injuries are a frequent presentation to the ED, with many of these being low risk for complications, such as non-displaced greenstick fractures, buckle fractures or soft tissue injuries. The current pragmatic reference standard for diagnosis is 2-view forearm x-rays, reported by a radiologist, which is also utilised in routine follow-up outpatient appointments to monitor progress. In most urban district centres, radiology services are readily available, including after-hours access, and are generally a quick process but can be delayed by more urgent cases [10]. However, in regional or austere settings, radiographic services can be less available, leading to potential delay in management or unnecessary transfers to larger centres [32]. POCUS is readily available in many prehospital and hospital settings and creates the opportunity to evaluate patients at the time of clinical review [33]. Validated clinical decision rules have demonstrated to prospectively reduce x-ray imaging by up to 19% with high sensitivity but lacking specificity [34].

POCUS has the potential to expedite the management decisions for children with paediatric distal forearm injuries including normal findings or a buckle fracture [10]. In these situations, patients can be safely discharged, in a wrist splint if a diagnosis of buckle fracture is made. It has been demonstrated POCUS can accurately diagnose paediatric distal forearm fractures when compared to x-rays when used by a variety of healthcare practitioners [10, 12–14]. However, no trial to date has demonstrated POCUS in this context to be non-inferior to x-ray imaging in terms of functional outcomes, and assessed its effect on cost-effectiveness, satisfaction, pain, complications, length of stay and diagnostic accuracy.

In particular, if POCUS is non-inferior in terms of functional outcome, then decisions on imaging modality will be primarily made based on cost, patient preference and pain. As such, our secondary outcomes will provide important insights for clinicians to make an informed decision on imaging modality. Additionally, avoidance of ionising radiation is of particular importance in the paediatric population where the ALARA principle is paramount [11]. If it can be established in this trial that POCUS is a suitable replacement for radiographs for forearm injuries, it could pave the way for further research into other paediatric applications, further limiting unnecessary ionising radiation.

This trial will be the first RCT conducted for using POCUS in the diagnosis of paediatric forearm injuries. This sentinel study will inform on both functional outcomes, as well as the cost-effectiveness of this approach.

### Trial status

Protocol version 2.0 date 31 July 2020. Recruitment has not yet commenced but is expected to commence in September 2020 and be completed by 2023.

## Data Availability

The datasets used and/or analysed during the current study will be available from the corresponding author on reasonable request and after requestees meet criteria stipulated by the approving ethics committee.

## Abbreviations

ALARA: As low as reasonably achievable
ANZCTR: Australian and New Zealand Clinical Trial Registry
CHU9D: Child Health Utility 9D
CI: Confidence interval
CT: Computerised tomography
ED: Emergency department
FPSR: Faces Pain Scale Revised
HREC: Human Research Ethics Committee
LOS: Length of stay
MRI: Magnetic resonance imaging
NP: Nurse practitioner
PCCRG: Paediatric Critical Care Research Group
POCUS: Point-of-care Ultrasound
PROMIS: Pediatric Upper Extremity Short Patient-Reported Outcomes Measurement Information System
QALY: Quality-adjusted life year
QOL: Quality of life
RCT: Randomised controlled trial
